# Age-Friendly Environment, Social Support, Sense of Community, and Loneliness of Korean Adults

**DOI:** 10.1093/geroni/igaa057.1533

**Published:** 2020-12-16

**Authors:** Miri Kim, Soondool Chung

**Affiliations:** Ewha Womans University, Seoul, Republic of Korea

## Abstract

Abrupt industrialization, urbanization, and the emergence of the nuclear family have contributed to the increase of single-person households, placing an emphasis on individualization in society. Because of such changes, more people are living alone and feeling lonely, alienated from society and family. This research examined the association between loneliness and age-friendly environment, a community equipped with policies, settings, services, and structures that promote active ageing and aging-in-place, with the mediating roles of social support and sense of community. The study analyzed a total of 590 respondents aged 45 and above using data from the age integration survey conducted by the OO Research Institute of South Korea. Structural equation modelling and the bootstrapping method were applied. Age-friendly environment was positively associated with social support (β=0.310,p=0.000) and sense of community (β=0.486,p=0.000). Social support was negatively associated with loneliness (β=-0.195,p=0.000). The full mediation effect of social support was observed in the pathway from age-friendly environment to loneliness (95% CI = [– 0.112, −0.028]). Social support was fundamental in lowering loneliness, and age-friendly environment could only reduce loneliness by following a pathway that enhances social support. In other words, despite how well an age-friendly environment is established, it would not decrease an individual’s loneliness unless it also enhances social support level. This finding sheds importance in maintaining satisfactory relationships that enable sharing of feelings, and fulfilling emotional and social needs in lowering loneliness. Measures to increase social support level within community are suggested.

